# Triple touch sperm immobilization vs. single touch sperm immobilization in ICSI - a randomised trial

**DOI:** 10.1186/1477-7827-10-65

**Published:** 2012-08-29

**Authors:** An Velaers, Goedele Paternot, Sophie Debrock, Thomas D’Hooghe, Carl Spiessens

**Affiliations:** 1 Leuven University Fertility Centre, UZ Gasthuisberg, Campus Gasthuisberg, Herestraat 49, 3000, Leuven, Belgium

**Keywords:** ICSI, Sperm immobilization, Immobilization techniques

## Abstract

**Background:**

Although different techniques for sperm immobilization have been described, their value has not been assessed in an adequately powered randomized study. The aim of this study was to compare two types of sperm immobilization methods prior to ICSI and to test the hypothesis that triple touch immobilization (TTIm) would lead to a higher (5% -65% up to 70%) fertilization rate (FR) than single touch immobilization (STIm).

**Methods:**

A total of 3056 metaphase II (MII) oocytes, from 290 patients, were randomly assigned to the STIm group (n = 1528 oocytes; 145 cycles) or to the TTIm group (n = 1528 oocytes; 138 cycles). A total of 1478 oocytes (STIm group) and 1476 oocytes (TTIm group) were used in the statistical analysis. The primary outcome variable was FR. Secondary outcome variables included: number of good quality embryos (GQE) on day 2 and day 3, implantation rate (IR) and implantation with foetal heart beat rate (FHB). Statistical analysis was done using the Fisher Exact test with a significance level of 0.05.

**Results:**

The results showed no differences in FR between both groups. The proportion of good quality embryos on day 3, was significantly higher in the STIm group (37.5%) compared to the TTIm group (31.8%; p = 0.02).

**Conclusions:**

In this RCT, the hypothesis that the post-ICSI FR would be higher after TTIm than after STIm was not confirmed and the number of good quality embryos on day 3 was significantly lower in the TTIm group than in the STIm group. These data suggest that more ‘aggressive’ TTIm technique has no advantages compared to the STIm technique.

## Background

Sperm plasma membrane damage has been described as a necessary process prior to intracytoplasmic sperm injection (ICSI) [1-4], as it plays a key role in the oocyte activation caused by the spermatozoon [3,4]. However, it is not fully understood to which extent sperm plasma membrane damage [3] is needed for adequate oocyte activation in the context of Assisted Reproductive Technology (ART) treatments.

Since the introduction of ICSI a number of studies have been conducted to evaluate the immobilization of spermatozoa prior to ICSI as a method to induce sperm plasma membrane damage before ICSI. Immobilization induces permeabilization of the sperm membrane and enhances subsequent nuclear decondensation [3]. Although the use of motile sperm cells instead of immobilized sperm has been promoted in the past [5], most studies agree that the use of immobilized sperm is necessary to provoke the processes needed prior to fertilization [1-4,6].

Different immobilization techniques can be used to induce sperm membrane permeabilization. The conventional method for immobilization consists of compressing the tail of the spermatozoon against the bottom of a dish with a micro injection pipette until a clear bend is visible [4]. More aggressive mechanical techniques include: permanently crimping the tail in the mid-piece region [4], cutting the tail below the mid-piece region [7], cutting halfway between the head and the tip of the tail [1] and dissecting the tail at the tip [8]. Finally, the application of lasers [9-11] or piezo-pulses [12] to sperm tails can induce sperm immobilization prior to ICSI.

When compared to the standard method, higher post ICSI fertilization rates for ejaculated spermatozoa have been reported after more aggressive mechanical [1,2,4,7] or piezo-pulse induced [12] sperm immobilization, but not after cutting the tail of the spermatozoon at different places [6] or applying laser-induced (non-contact 1.48-μm diode laser) sperm immobilization [10,11] techniques for sperm immobilization.

Although different techniques for sperm immobilization before ICSI have been described, their value has not been assessed in an adequately powered randomized study. The majority of studies mentioned above were published more than 10 years ago in selected groups, i.e. men with oligoterato- or oligoasthenozoospermia [1,7] and using a low number of oocytes (about 500 oocytes) except for 1 study (6419 oocytes) [4]. No subsequent study has been conducted recently, including a large number of oocytes in a patient population representative for routine clinical practice. As biological plausibility [3] and observational literature data support the possibility that more sperm plasma membrane damage may lead to higher fertilization rate after ICSI, the aim of our study was to test the hypothesis that triple touch sperm immobilization (TTIm) would lead to a higher fertilization rate (FR) after ICSI than single touch sperm immobilization (STIm). The hypothesis is that the damage induced to the mid-piece region would imply a more successful outcome in ICSI, due to the fact that the mitochondria are tightly packed and located only in the mid-piece region [13].

## Methods

### Patients

All ART ICSI cycles between April 2009 and January 2010 were eligible for our randomized study, except ICSI cycles combined with pre-implantation genetic diagnosis (PGD), or ICSI cycles using either immotile sperm from fresh ejaculate, or sperm from testicular biopsies. Patients received treatment with ICSI mostly for the indication of severe male factor infertility (87%) (See Table 1). The ovarian stimulation and egg retrieval protocols have been described in detail before [14].

**Table 1:** Patients’ and cycles characteristics of the study objects

**Patient characteristics**	
Mean Age Female (±SD)	31.68 (±4.47)
Mean Age Male (±SD)	34.58 (±5.96)
Cause of subfertility	
* N Tubal factor (%)*	53 (11)
* N Ovulation (%)*	68 (15)
* N Endometriosis (%)*	51 (11)
* N Implantation (%)*	14 (3)
* N Other (%)*	13 (3)
* N No female indication (%)*	260 (57)
* N Male factor (%)*	398 (87)
Total Motility Count (Median/Min/Max)(x 10^6^)	4.28 (2.00/< 0.01/40.50)

### Semen collection and preparation

Briefly, semen samples were processed following the SOPs (Standard Operating Procedures) of the Leuven University Fertility Centre (LUFC).

Semen samples were collected by masturbation and prepared after liquefaction (20 minutes at 37°C) [15,16]. The samples were prepared on a three layer Isolate gradient (100%, 70%, and 50%) (Isolate^TM^, Irvine Scientific, Santa Ana, USA) and washed with HEPES-buffered culture medium (Gynemed, Lensahn, Germany).

### ICSI procedure

The micromanipulation procedure was performed on the lid of a Petri dish (Nunc, Thermo Scientific, Roskilde, Denmark) using droplets (20 μl) of HEPES-buffered culture medium (Gynemed, Lensahn, Germany), incubated at 37°C in 5% CO2 in air. To avoid evaporation, droplets were covered with mineral oil (GM, Gynemed, Lensahn, Germany). Prior to ICSI, a small amount of spermatozoa (± 5 μl) was placed in a polyvinylpyrrolidone droplet (GM PVP, Gynemed, Lensahn, Germany). Injection pipettes (COOK Cook, Brisbane, Australia/Humagen Origio, Malov, Denmark) and holding pipettes (COOK Cook, Brisbane, Australia) were used for micromanipulation. Micromanipulation (at 37°C on a heating plate) was performed using an inverted microscope with modulation contrast using a 200x magnification.

### Sperm immobilization and randomization

**Figure 1:**
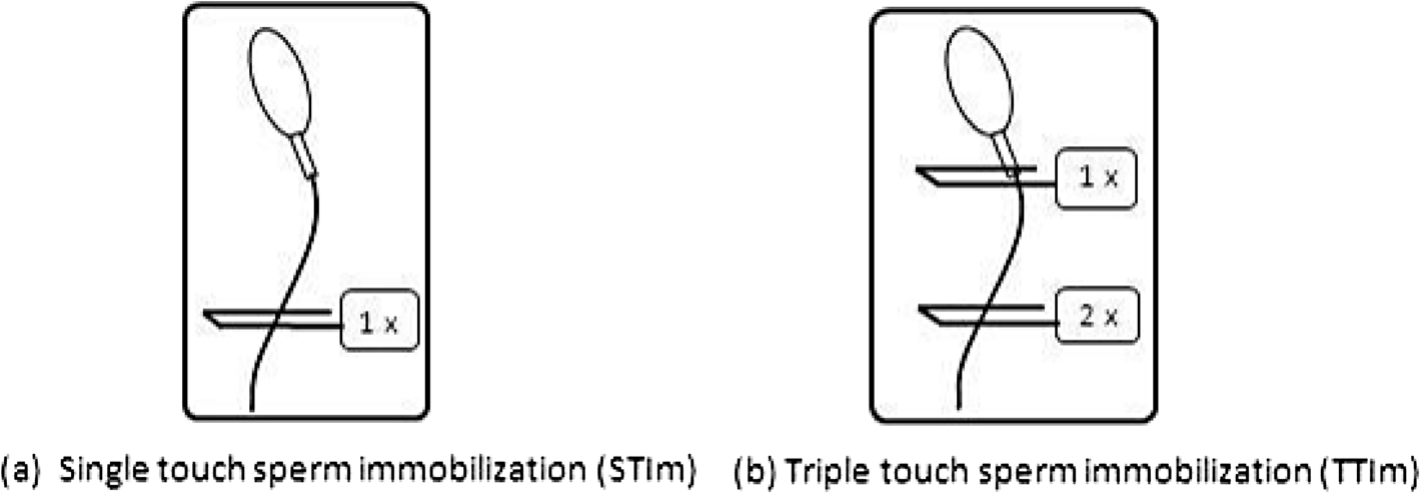
**Sperm immobilization.** (**a**) Standard procedure STIm : pressing the tip of the tail against the lid of the dish with a microinjection pipette until a clear small bend in the middle of the flagellum is visible (**b**) More aggressive procedure TTIm: based on application of the standard procedure twice and compression of the sperm mid-piece once.

Randomization was performed at the time of oocyte denudation. In case of an odd number of MII oocytes, the last oocyte was randomized using a blinded envelope system. Sibling MII oocytes were randomly assigned to the STIm or TTIm technique. In the STIm group, immobilization was performed by pressing the tail of the spermatozoon to the bottom of the dish with the injection pipette and then quickly withdrawing this pipette until a clear bent in the middle of the flagellum was observed [2,17,18] (see Figure 1). In the TTIm group the spermatozoon was immobilized three times: twice on the tail (STIm technique applied twice) and once by compressing the mid-piece [8,19] (see Figure 1). In case of not performing the STIm or TTIm technique in a correct manner (further kinetic movement of the sperm tail), another spermatozoa was selected and permanently immobilized for the injection in “either” groups.

### Fertilization control and embryo evaluation, embryo transfer

After injection the oocytes were washed in droplets of culture medium and placed into a new culture dish with culture medium, divided into two groups (co-cultured) and incubated overnight.

For both the STIm and TTIm group, fertilization control was performed 16 to 20 hours later on a stereomicroscope on 37°C. Oocytes showing 2 pronuclei (2PN) were identified as normally fertilized and were cultured individually, the presence of 1 (PN) or 3 (3PN) pronuclei was considered as abnormally fertilized.

On day 2 and day 3, embryo quality was assessed using embryo development and morphology. The number and size of blastomeres and the percentage of fragmentation was evaluated. A good quality embryo (GQE) on day 2 was defined as a 4- cell stage embryos with less than 25% fragmentation and equally or slightly unequally sized blastomeres and on day 3 (7-, 8-, or 9- cell stage embryo with less than 25% fragmentation and equally or slightly unequally sized blastomeres). On day 5 the blastocyst stage was evaluated based on the presence of the inner cell mass, the trophectoderm layer, the blastocoel and the degree of expansion as described before [20]. A blastocyst with a blastocoel completely filling the embryo, a tightly packed inner cell mass and a trophectoderm with many cells forming a cohesive epithelium was defined as a good quality blastocyst at day 5.

A single (SET) or double (DET) embryo transfer was performed, independent from the immobilization technique, on day 2 (N = 47), day 3 (N = 404) or day 5 (N = 8) according to the transfer policy decided by the gynaecologist (based on the Belgian law of July 2003) at the start of the cycle and the number of fertilized oocytes on day 1.

Clinical implantation and pregnancy results were obtained for the STIm versus TTIm method and also for mixed embryo transfers. 

### Outcome variable and power calculation

This study was performed on sibling oocytes to test the hypothesis that the fertilization rate (FR) per oocyte would be higher in the TTIm group (70%) than in the STIm group (65%) based on the average FR in our fertility centre during 2008.

The primary outcome variable was the FR, defined as the number of normally fertilized oocytes over the total number of MII oocytes and over the total number of successfully injected MII oocytes. Therefore a total of 1528 mature oocytes were required in each group assuming a power of 0.80, α = 0.05, resulting in a total of 3056 MII oocytes in the whole study.

Secondary outcome variables included: number of good quality embryos (GQE) on day 2 and day 3, utilisation rate (number of embryos available for embryo transfer and cryopreservation over the total number of normally fertilized oocytes) (UR), implantation rate per embryo transferred (IR) [21] and implantation rate with foetal heart beat per embryo transferred (FHB). Statistical analyses were done using the Fisher Exact test with a significance level of 0.05.

## Results

**Figure 2:**
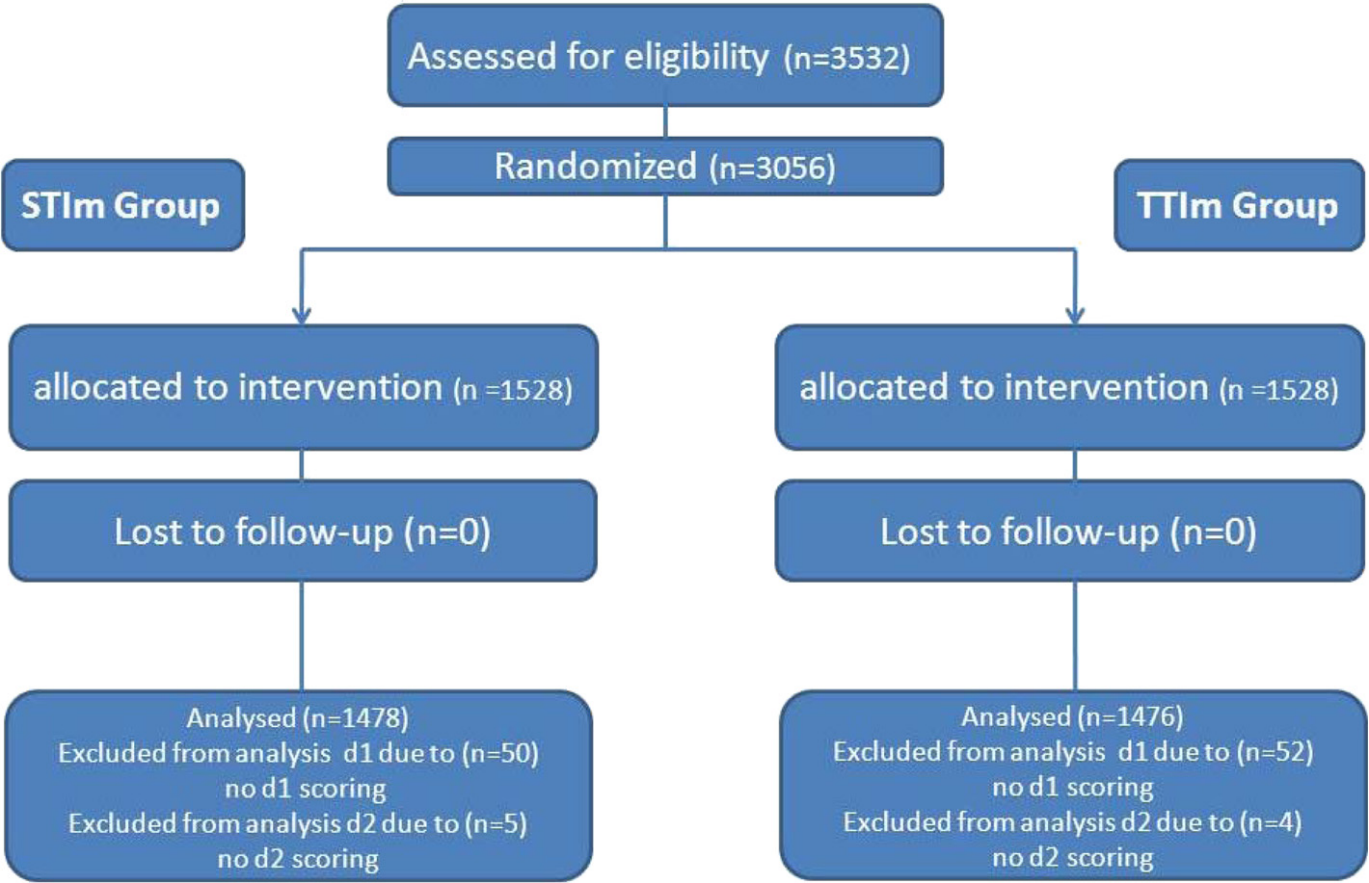
Flow diagram of randomization of oocytes prior to ICSI.

This randomised study, performed between April 2009 and January 2010 in 290 ICSI cycles, was done with a total number of 3532 oocytes and after removal of the cumulus cells, 3056 MII oocytes were available for ICSI. After randomisation, 1528 MII oocytes (145 cycles) were assigned to the STIm group and 1528 MII oocytes (138 cycles) to the TTIm group. Oocytes (102MII) were excluded from the analysis due to missing data on day 2 or day 1, if they were excluded for analysis for day 2, than they were automatically excluded for day 1. Respectively 50 oocytes (STIm group) and 52 oocytes (TTIm group) were excluded from the analysis (see Figure 2). Consequently, 1478 MII for the STIm group and 1476 MII for the TTIm group were available for analysis. Mean age (±SD) was 31.7 (±4.5) and 34.6 (±6.0) for female and male partner respectively.

Both groups were comparable with respect to the fertilization rate per injected MII oocyte (STIm: 67.1% vs TTIm: 66.7%), fertilization rate per successfully injected MII oocyte (STIm: 74.8% vs 75.8%), percentage of abnormally fertilized oocytes (STIm: 6.7% vs TTIm: 6.1%) and the embryo utilisation rate (STIm: 51.7% vs TTIm: 47.2%) (UR) (Table 2). The number of good quality embryos on day 3 was significantly higher in the STIm group compared to the TTIm group (STIm: 37.5% vs TTIm: 32.2%; p 0.02). The embryo quality on day 2 (STIm: 33.0% vs TTIm: 31.8%) and day 5 (STIm: 17.2% vs TTIm: 20.7%) was comparable in both groups, although only a small number of day 5 blastocysts were included (Table 2). The pregnancy outcome as measured by IR per embryo transferred (STIm: 31.0% vs TTIm: 23.9%) and IR with foetal heart beat per embryo transferred (STIm: 27.6% vs TTIm: 23.2%) was comparable in the STIm group and the TTIm group (see Table 3).

**Table 2:** Results for embryological data

**Total number of oocytes**	**3532**		
**Total number of mature** **oocytes = total number of injected oocytes**	**3056**		
**Outcome variable**	**STIm -group**	**TTIm-group**	**P-value**
% Fertilization rate/mature oocytes injected (n)	67.1 (992/1478)	66.7 (984/1476)	0.81
% Fertilization rate/successfully injected oocytes (n)	74.8 (992/1326)	75.8 (984/1298)	0.56
**Parameter**			
% 1pn/mature oocytes injected (n)	4.5 (67/1478)	3.5 (51/1476)	0.16
% 3pn/mature oocytes injected (n)	2.2 (32/1478)	2.6 (38/1476)	0.55
N GQE d2/total n embryos on d2	326/987	312/982	
N GQE d3/total n embryos on d3	348/929	300/932	
N GQE d5/total n blastocysts on d5	5/29	6/29	
% of good quality embryos on d2 (n)	33.0 (326)	31.8 (312)	0.56
% of good quality embryos on d3 (n)	37.5 (348)	32.2 (300)	0.0172
% of good quality embryos on d5 (n)	17.2 (5)	20.7 (6)	>0.9999
% Utilisation rate (n)	51.7 (513)	47.2 (464)	0.24

“Parameters evaluated in both groups (STIm versus TTIm). The number of good quality embryos on day 3 was significantly different between both groups; the other characteristics were equal in both groups.”

**Table 3:** Results for clinical data

**Parameter**	**STIm -group**	**TTIm-group**	**P-value**
N embryos transferred	145	138	
% Implantation/embryo transferred (n)	31.0 (45/145)	23.9 (33/138)	ns
% Implantation with featal heart beat (FHB)/embryo transferred (n)	27.6 (40/145)	23.2 (32/138)	ns
N embryos transferred in SET	63/145	63/138	
N embryos transferred in cycli with at least 2 embryos transferred	82/145	75/138	

Statistical analysis was performed using Fisher’s exact test.

## Discussion

In this RCT, the hypothesis that the post-ICSI FR would be higher after TTIm than after STIm was not confirmed. Furthermore, the proportion of good quality embryos on day 3 (secondary outcome variable) was significantly lower in the TTIm group than in the STIm group. Collectively, these data suggest that more ‘aggressive’ TTIm technique has no advantages compared to the STIm technique before ICSI. To the best of our knowledge, our study represents the first randomized study in a large patient population, with FR as primary outcome variable, based on a priori power calculation and a sufficiently high number (n = 2954) sibling MII oocytes available for analysis.

The results of our study confirm the data (similar FR and embryo quality on day 2) from another RCT [8], but including only 205 MII oocytes to compare 3 different immobilization techniques: compressing the mid-piece (comparable to the TTIm technique used in our study), cutting the tail at the mid-portion and dissecting the tail of the spermatozoon at the tip. However, the results of our study are in disagreement with the increased FR after ICSI reported in non-randomized retrospective case control studies [1,2,4,7] using other mechanical, aggressive sperm immobilization techniques in different types of spermatozoa (immotile, thawed, epididymal), when compared to the standard immobilization method. Furthermore, when compared to STIm, we did not confirm improved outcome after TTIm for previously reported secondary outcome variables like: a significant decline in degenerated oocytes [7] or a decrease of the number of 1PN oocytes [1].

The results of our study don’t allow us to draw conclusions on the underlying mechanisms. A possible consideration could be that the amount of damage needed to immobilize a motile spermatozoa is not depending on the number of strokes (STIm vs TTIm) but rather on the region (mid-piece) where we immobilized the sperm tail. We have to keep in mind that other reactions such as hyperactivity due to the mitochondria at the site of fertilization might have an influence on the fertilization rate [13]. In addition, we cannot exclude, based on our results, that no additional damage occurs to the centrosomes of the spermatozoa after both immobilization techniques. The importance of centrosomes has been studied in the last few years, however the exact comprehension of the mechanisms remains unclear [22].

## Conclusions

In conclusion, this randomized trial showed that, when compared to STIm, TTIm did not result in a higher post-ICSI fertilization rate (primary outcome) and was associated with a lower number of good quality embryos on day 3 and with similar pregnancy rates (secondary outcomes), suggesting that the more ‘aggressive’ TTIm technique has no advantages compared to the STIm technique before ICSI.

## Competing interests

The authors declare that they have no competing interests.

## Authors’ contributions

VA and SC designed the study. VA and PG analysed and interpreted the data. These authors draft the paper and approved the final version. DS, D’HT and CS interpreted the data and revised the paper critically for intellectual content and approved the final version. All authors read and approved the final manuscript.
